# The Agglutination of Tumour Cells In Vitro by Sera from Tumour Patients and Pregnant Women

**DOI:** 10.1038/bjc.1964.11

**Published:** 1964-03

**Authors:** C. Tal, T. Dishon, J. Gross

## Abstract

**Images:**


### THE AGGLUTINATION OF TUMOUR CELLS *IN VITRO* BY SERA FROM TUMOUR PATIENTS AND PREGNANT WOMEN

C. TAL, T. DISHON AND J. GROSS

*From the Department of Experimental Medicine and Cancer Research, and the Laboratory of Experimental Neurology, The Hebrew University, Hadassah Medical School, Jerusalem, Israel*

Received for publication December 7, 1963

IN the course of study on the possibility of autoimmune mechanisms against tumour, the level of circulating antibodies to the autologous tumour cells was estimated by cell agglutination titres according to a technique previously developed in this laboratory (Pikovski, Tal, Schlesinger and Margoliash, 1957). It was soon found that cross agglutination occurred. It appeared that the sera from tumour patients contained a factor (AF) which could cause agglutination of, and be adsorbed by, tumour cells from a variety of sources. This paper represents an analysis of this phenomenon.

### MATERIALS AND METHODS

### *Cell preparation*

Cell suspensions were prepared from some human tumours, several normal human tissues and some mouse tumours by a previously described technique (Pikovski, Tal, Schlesinger and Margoliash, 1957). Suspensions of tissue cultures of HeLa cervical carcinoma, KB buccal carcinoma and Chang normal liver cells were also used. The preparation of the tissue culture suspensions is given in the following detail using HeLa cultures as the example.

HeLa cultures were grown in Eagle's lact-yeast medium plus 15 per cent of normal human serum and horse serum, respectively, in milk bottles. After 7–8 days of growth, the medium was pipetted off, and the cell layer washed with 10 ml. of warm (37° C.) phosphate-buffered saline (PBS). The PBS was replaced by 10 ml. of 0·02 per cent Ethylenediamine Tetracetic acid (EDTA) in PBS devoid of Ca and Mg, and the culture kept at 37° C. for 15–20 minutes. This procedure loosened the cells from the wall of the culture bottle and the EDTA cell suspension was transferred to a test tube. Clumps of cells were dispersed by repeated pipetting with a fine Pasteur pipette, taking care to avoid foaming. The suspension was then centrifuged at 500 r.p.m. for 5 minutes. The supernate was carefully removed so as not to disturb the cell pellet. The cells were then resuspended in 8 ml. of warm PBS and centrifuged as above. The washed cells were counted and suspended in sufficient normal saline to give a concentration of $4 \times 10^6$ cells per ml.

### *Agglutination procedure*

Sera were diluted fourfold with 0·2 M phosphate buffer (pH 7·0). The pH of the system was critical. $2 \times 10^5$ Cells were added to 0·4 ml. of serum dilutions.

The contents were mixed and the tubes incubated for 2 hours at 37° C. They were then kept at 4° C. for 16 hours and then returned to 37° C. for a further incubation period of 3 hours. The result was considered positive if all the cells of the tube clumped into a single pellicle which did not break up when shaken gently. This is shown in Fig. 1. The greatest dilution showing this result was taken as the titre. In some sera agglutination did not begin to appear till there was some dilution of the serum. This was analagous to the zone effect for antigen antibody precipitation where in a zone where the antigen is present in excess the precipitation is less than maximum. In the mass survey of sera (Table I) the specimens were coded and then given to the testers, who were therefore unaware of the origin of the serum.

*Adsorption and elution procedure*

One ml. of serum was added to 2 ml. of washed packed cells. The tube was stoppered and mixed. The suspension was incubated at 37° C. for 2 hours and then left overnight at 4° C. After centrifugation the supernatant was tested for the presence of the agglutinating factor.

The cells or cell fractions after they had adsorbed the agglutinating factor from the serum were washed twice with normal saline and then mixed with an appropriate volume of 3 per cent sodium chloride. The latter was used as a reagent to remove the adsorbed protein from the cells or cell fractions. This mixture was incubated 2 hours at 37° C. and left over night at 4° C. It was then centrifuged and the supernate concentrated by dialysis against 25 per cent polyvinylpyrrolidone in normal saline to the desired concentration.

*Serum fractionation procedures*

Serum pools from normal individuals and those suffering from neoplastic diseases were separated into 3 fractions, i.e. albumin plus alpha globulin, beta globulins and gamma globulins by the method of Lever, Gurd, Uroma, Brown, Barnes, Schmid and Schultz (1951). These were tested for agglutination of tumour cells. A further fractionation was carried out. Serum pools were treated with one third their volume of saturated $(NH_4)_2SO_4$. The resulting precipitate was dissolved in distilled water and dialysed against 0·9 per cent NaCl. The solution was then treated with an equal volume of 3·6 M $(NH_4)_2SO_4$ the resultant precipitate was dissolved in 0·9 per cent NaCl and dialysed against distilled

EXPLANATION OF PLATE

FIG. 1.—This figure illustrates the appearance of the control suspension of tumour cells (left) and the agglutinating effect (right) of tumour sera on such suspensions. The formation of such a single pellicle was taken as the criterion for a positive agglutination reaction. The maximum serum dilution giving such a reaction was taken as the agglutination titre.

FIG. 2.—The figure illustrates the results of immunoelectrophoresis of the beta globulin fractions from normal and tumour sera when reacted with a rabbit anti-serum against the fraction from tumour sera. The anode is located to the left and the cathode correspondingly to the right in these preparations.

The normal serum fraction (A) shows 3 precipitation lines while the tumour serum fraction (B) shows at least 6 distinct components. The latter was absorbed with placental tissue from which it was then eluted. The eluate (C) shows the presence of 3 protein components. The line indicated by the arrow was prominent in all tumour sera tested.

1

2

Tal, Dishon and Gross.

water (pH7). A precipitate formed which was then dissolved in 0·9 per cent NaCl or Hank's solution. This solution prepared from tumour sera, contained the agglutinating factor. It was further analysed by microimmunoelectrophoresis (Scheidegger, 1955) using rabbit anti-sera against the fraction and against whole sera.

*Cell fractionation procedures*

As a preliminary fractionation, cells were disrupted and separated by centrifugation into precipitate and supernatant fractions. The detailed procedure is as follows—2 ml. of packed tumour cells were suspended in 5 ml. of 0·9 per cent NaCl. The suspension together with 7 g. of glass powder was shaken in a Nossal shaker in the cold for 30 seconds. After standing at room temperature for ½ hour to eliminate the glass, the supernatant was removed and centrifuged at 10,000 r.p.m. for ½ hour. The precipitate was washed with saline 3 times by centrifugation. The washings were discarded. The precipitate was tested for its ability to absorb the agglutinating factor from serum. The supernatant was tested for possible precipitation activity with normal or tumour sera.

Since the precipitate was found to remove the agglutinating capacity of positive serum, a further fractionation was carried out (Goebel, Binkley and Perlman, 1945). Cell suspensions or tissue homogenates were washed with saline to remove all blood or medium. The material was then treated with about 4 volumes of acetone and this procedure was repeated twice.

The precipitate was then air dried. The dry material was then extracted with about 4 volumes of ether and air dried. Thirty ml. of undiluted pyridine per gram of dry residue were added and the mixture homogenized. The pyridine suspension was then incubated at 37° C. for 1 hour and left at room temperature overnight. The supernate was removed and the residue again extracted with 15 ml. of pyridine per gram. The supernatants were pooled and dialysed against distilled water at room temperature until free of pyridine odour. A fine precipitate formed, which on the addition of 10 volumes of acetone, sedimented readily by centrifugation at 3000 r.p.m. for 5 minutes. The precipitate was air dried and constituted about 3 per cent of the dry weight of the starting material. It was found to adsorb the agglutinating factor.

### RESULTS

*Incidence of AF in human sera*

A first step in this study, was to determine if there was any association between serum agglutinating capacity and the presence of neoplasia in the serum donor. HeLa cells were used as the routine test object. Sera were obtained from the following sources.

(*a*) 120 patients with histologically proven tumour, attending the tumour clinic for ambulatory treatment.

(*b*) 237 typing samples of blood, taken from consecutive blood donors at the blood bank.

(*c*) 52 patients consecutively admitted to one of the internal medicine services at the university hospital. These individuals suffered from a variety of chronic diseases, about 15 per cent of the cases however were diagnosed as acute myocardial infarction. In none of the patients was there a suspicion of cancer.

(*d*) 12 women at delivery and at the same time samples of cord blood from their offspring.

The results obtained are given in Table I.

TABLE I.—*Mass Survey of Sera*

| Source of serum | Age of donor Mean and range | Number tested | Number positive | % Positive |
|---|---|---|---|---|
| Normal individuals | | | | |
| Blood bank | 31 years (18–56) | 237 | 31 | 13 |
| Term pregnancy | — | 12 | 12 | 100 |
| Cord blood | — | 11 | 1 | 9 |
| Diseased individuals | | | | |
| Non-malignant | 47 years (15–80) | 51 | 8 | 16 |
| Malignant disease | 51 years (17–79) | 120 | 108 | 90 |

The frequency of agglutination is high in sera from patients with malignant disease and in sera from pregnant women.

The age distribution in both groups of diseased individuals is similar but the incidence of AF in the sera of the non-malignant group is significantly lower ($P < 0{\cdot}001$ by the Chi square test) than in sera of individuals with cancer. Thus the presence of AF does not appear to be a concomitant of either chronic disease or of ageing.

An additional difference is seen in the histogram of titres obtained in each of the test groups (Fig. 3). In cancer sera the modal AF titre is higher than in either of the two types of controls.

The effect of tumour site on the agglutination reaction is shown in Table II. The percentage of positives is roughly equivalent for all types. The numbers are too small to draw any definite conclusions but there is an indication that there are fewer positive reactions in the sera of patients suffering from tumours of connective tissue origin.

TABLE II.—*Serum HeLa Cell Agglutination Activity According to Tumour Location*

| Tumour type | Number sera | Number positive | % |
|---|---|---|---|
| Mammary | 50 | 48 | 96 |
| Female genital | 22 | 18 | 82 |
| Gastro-intestinal | 12 | 11 | 92 |
| Respiratory tract | 9 | 8 | 89 |
| Connective tissue | 12 | 9 | 75 |
| Miscellaneous | 15 | 14 | 94 |

*The nature of the serum agglutinating factor (AF)*

The separation of serum pools into 3 large fractions was carried out. Only the beta globulin fraction of the tumour sera contained the agglutinating factor. The remaining fractions of tumour sera and all fractions of normal sera were negative for AF.

By further fractionation, using a method designed for the isolation of beta globulins, a preparation was obtained which was positive for AF. A rabbit anti-serum was prepared against the fraction and used for its immunoelectrophoretic analysis. The results are shown in Fig. 2. The beta fraction from normal serum showed about 3 components (Fig. 2A), while the AF-positive beta fraction usually contains at least 6 antigenically distinct components (Fig. 2B). This mixture was absorbed with placental preparation and subsequently eluted with 3 per cent NaCl. The eluted material, causing cell agglutination, showed the presence of at least 3 protein components (Fig. 2C). Fifteen tumour serum beta fractions and 11 normal serum beta fractions were analysed individually by immunoelectrophoresis. Twelve of the tumour sera preparations showed more

FIG. 3.—A comparison of the agglutination-titres found in the sera from patients with proven malignancy and the sera from the two control groups.

lines than the normals and all tumour preparations showed the two prominent lines to the cathodal side of the well (see arrows Fig. 2B and 2C). The samples subjected to immunoelectrophoretic analysis were all of the same protein concentration, i.e. 15 mg. per ml. Absorption of beta fraction from tumour sera with an anti-serum against the fraction from normal serum removed all lines.

*Cell factors involved in the agglutination reaction*

A number of normal and tumour cell suspensions were tested for agglutinability by tumour sera. Table III shows that, with the exception of KB cells, all the tumour cells tested, were agglutinated. The normal tissues tested did not show this reaction. While there seems to be a specificity for the reaction with tumour cells, it is possible that other types of normal cells might show agglutination.

A similar pattern was found in relation to the absorption of AF from sera by various cell types. These results are shown in Table IV, AF was removed from 1 ml. serum by as little as 0·04 ml. of packed cells from various tumour cell pre-

TABLE III.—*The Agglutinability of Various Cell Types With Tumour Sera*

| Cell type | Agglutinability |
|---|---|
| Normal tissues— | |
| Human liver cell suspensions | 0 |
| Human liver cell in tissue culture (Chang) | 0 |
| Human kidney cell suspension | 0 |
| Tumour tissues— | |
| Human mammary carcinoma | + |
| Human colon bladder carcinoma | + |
| Human gall bladder carcinoma | + |
| Human cervical carcinoma in tissue culture (HeLa) | + |
| Human buccal carcinoma in tissue culture | 0 |
| Mouse mammary carcinoma (RIII) | + |
| Mouse mammary carcinoma (C3H) | + |
| Mouse ascites tumour (Ehrlich) | + |

parations and human placental homogenate. Normal human liver, kidney or heart showed no such activity even when as much as 2 ml. of packed tissue was tested for adsorption from 1 ml. of serum.

TABLE IV.—*Specific Absorption of the Agglutinating Factor in the Serum of Cancer Patients*

Procedure—The active sera were mixed with about 6 × $10^6$ cells or the equivalent of tissue homogenate, incubated at 37° C. for 2 hours, at 4° C. for a subsequent 16–18 hours, and then centrifuged. The supernate was then tested for agglutinating power.

| | Agglutination titre | | | | | |
|---|---|---|---|---|---|---|
| Cell type | Serum No. 119 | Serum No. 208 | Serum No. 71 | Serum No. 307 | Serum No. 412 | Serum Pool No. 4 |
| Non-absorbed control | 1 : 64 | 1 : 128 | 1 : 16 | 1 : 128 | 1 : 256 | 1 : 64 |
| RIII mouse mammary carcinoma | 0 | — | 0 | — | — | — |
| HeLa human cervical carcinoma | 0 | — | — | — | — | — |
| Kidney homogenate human | 1 : 64 | 1 : 128 | 1 : 16 | 1 : 128 | 1 : 256 | 1 : 64 |
| Liver ,, ,, | 1 : 64 | 1 : 128 | 1 : 16 | 1 : 128 | 1 : 256 | 1 : 64 |
| Heart ,, ,, | 1 : 64 | 1 : 128 | 1 : 16 | 1 : 128 | 1 : 256 | 1 : 64 |
| Placenta ,, ,, | — | 0 | — | — | — | — |
| Ehrlich mouse ascites tumour | — | — | — | 0 | — | — |
| Human mammary carcinoma | — | — | — | — | — | 0 |

(—) Not done.

Crude fractionation was carried out of human mammary carcinoma, RIII mouse mammary carcinoma and Landschutz mouse ascites tumour into a fraction sedimentable at 10,000 r.p.m. and a supernatant. The washed sediments were found to absorb AF from tumour sera. The supernates were also tested with normal and tumour sera for possible immune precipitation reactions—none was found. This suggested the possibility that the absorbant might be cell wall material, e.g. lipo-mucopolysaccharide-protein-complex. A procedure used for extracting such substances from bacteria by means of pyridine (Goebel, Binkley and Perlman, 1945) was empirically applied to 2 mouse tumour tissues (Landschutz mouse ascites, RIII mouse mammary carcinoma), a human mammary carcinoma and to human placenta. In all cases a water-insoluble precipitate was obtained which absorbed the agglutinating factor from cancer sera. The absorbed material could be eluted by treatment with 3 per cent NaCl solution. The eluates caused agglutination of HeLa Cells. As to the nature of the pyridine-extractable component, rough analysis of such a preparation from human placenta shows the presence of a large amount of lipid (66 per cent) some protein (15 per cent) and the rest carbohydrate.

### DISCUSSION

Alterations in the serum of patients with cancer have been extensively investigated. These have been found primarily in the quantitative and qualitative composition of the serum proteins (reviewed by Winzler, 1953, and Petermann, 1961). Changes in tumour sera which may cause agglutination of tumour cells have not previously been reported. However there is a report by Saxen and Penttinen (1961) that of all fresh human sera added to tissue cultures of HeLa cells, 9 per cent cause clumping of the cells and a reduction in their viability. This effect requires fresh serum and can be removed by absorption with HeLa cells. Although the effect was observed in 40 per cent of the bloods of pregnant women at term it occurred in only 26 per cent of the cancer bloods tested (Saxen and Penttinen, 1962). At the present time it is uncertain whether this clumping phenomenon is analogous or related to the agglutination effect reported in this paper.

Some speculation is possible as to the nature of the serum agglutinating factor. Normal human sera have been shown to be cytotoxic to several types of tumour cells. The toxic factor is absorbed by tumour, placental and lymphatic tissue (Landy, Michael, Trapani, Achinstein, Woods and Shear, 1960 ; Ginsburg, Dishon, Bloch and Gross, 1961). This property is located in a serum protein fraction which is glycoprotein in nature and consists of a group of proteins migrating in the beta 2 region when subjected to immunoelectrophoresis (Dishon and Gross, unpublished). In fractionating serum from tumour patients, the agglutinating capacity was found *pari passu* with the cytotoxic activity described above. All attempts to separate the agglutinating and cytotoxic activities were so far unsuccessful. This localization of the agglutinating factor to the beta globulin is in accord with the finding of Takada, Saito, Tanino and Ebata (1962) that there is an additional line demonstrable in the beta 2 region in 81 per cent of all cancer sera tested, and in 10 per cent of sera from non-cancer patients. However the serum change reported here differs from the serum factor in pregnancy and malignancy studied by Rottino, Angers and Dool (1962) which has been localized in the alpha globulin region.

The proteins of normal and tumour sera fractions appear to differ mainly in their immunoelectrophoretic appearance. Thus the two major immunoelectrophoretic lines demonstrable in the agglutinating fraction of tumour serum appear to form longer arcs extending more toward the cathode than the normal. The beta fraction area from neoplastic or pregnant sera shows an additional concentric precipitation line which is not visualized in the normal beta serum fraction. Antigenically, both normal and tumour fractions are the same, since all the lines of the latter are removed by absorption with anti-serum against the beta fraction of normal serum. This absorption also removes the agglutinating and cytotoxic properties. The AF therefore may be due either to the enrichment of a normal constituent of the beta proteins and/or it may be a metabolic product of the normally circulating cytotoxic factor. This product could be the result of the metabolism of tumour or placental tissues themselves, or the activation of a normal metabolic mechanism by the presence of these tissues in the body. One such possibility is a partial proteolytic cleavage of the normal protein. In this connection it should be noted that Heremans (1960) found that treatment of beta 2 globulins with papain, *in vitro*, resulted in an immunoelectrophoretic pattern consisting of several precipitation lines in addition to the lines given by the native protein. The possibility that there might be *only* an enrichment of the normal beta fraction is unlikely since a fourfold concentration of normal beta fraction did not cause agglutination.

The possible mechanism of agglutination can now be examined. The serum protein probably responsible for the agglutination is absorbed onto the HeLa cells and differs from its counterpart in normal serum by being somewhat more positively charged. This is indicated by its greater migration toward the cathode in electrophoresis (see Fig. 2). A second point is the finding that the conditions for the test require a careful control of pH. Sera from all sources tended to cause HeLa cell agglutination if the pH of the incubation medium exceeded 7·5 and conversely no agglutination occurred when the environment pH was less than 6·8. This would suggest that agglutination is a reflection of changes in surface charge on the HeLa cell caused by the difference in charge of the adsorbed serum protein. Similar mechanisms have been demonstrated to occur in the case of the agglutination of red blood cells (Sachtleben, Ruhenstroth-Bauer, 1961). An additional factor involved, may be the level of seromucoid in the serum. Thus Sato, Amizuka and Sato (1962) have found that the non-specific agglutination of kieselguhr by human serum is quantitatively inhibited by increasing additions of human seromucoid. It is established that in cancer there is an elevation in serum seromucoid (Winzler, 1953). If this component plays a role in the HeLa cell agglutination system, it is possible that failure to obtain agglutination in some cancer sera (Table I) may have been due to an excessive elevation of this serum component.

### SUMMARY

It has been found that HeLa cells under appropriate conditions were agglutinated by 90 per cent of 120 sera from patients suffering from proven malignancy. On the other hand, only 16 per cent of sera from 51 patients suffering from non-neoplastic chronic disease, and 13 per cent of 237 normal bloods showed such an effect. Twelve bloods obtained from pregnant women at term all showed agglutination. A number of tumour cell suspensions were tested for agglutinability, and with the exception of KB (buccal carcinoma cells) all were positive. Suspensions of normal liver or kidney cells did not agglutinate. The agglutinating factor was absorbed from serum by cell suspensions from a number of human and animal tumours and from human placenta. Human kidney, liver or heart showed no such absorptive capacity. A partial isolation of the tumour cell factor responsible for the absorption was carried out. The fraction responsible for agglutination localized to the $\beta$ globulins of the serum protein spectrum. In tumour sera examined by immunoelectrophoresis, an additional line became obvious.

The efforts of the late Dr. E. Tennenbaum in providing continuous and copious supplies of HeLa cells were indispensable to this study, for this the authors are most grateful to her.

The authors are indebted to Professors A. Hochman and M. Rachmilewitz who kindly permitted access to the patients on their respective services. Thanks are due to Dr. J. Stein and Dr. G. Zajicek for taking and coding the blood samples.

The indispensable technical assistance of Miss Ahuva Hari, Mrs. Yaffa Ingster, Mrs. Miriam Schlesinger and Mr. Nissim Conforti is gratefully acknowledged.

This work was supported in part by an equipment grant from the Damon Runyon Fund and a grant from the Cancer Research Fund of Canadian Hadassah (Wizo).

### REFERENCES

GINSBURG, I., DISHON, T., BLOCH, M. AND GROSS, J.—(1961) *Proc. Soc. exp. Biol., N.Y.*, **107**, 235.

GOEBEL, W. F., BINKLEY, F. AND PERLMAN, E.—(1945) *J. exp. Med.*, **81**, 315.

HEREMANS, J.—(1960) 'Les Globulins Seriques du System Gamma ' Paris (Masson).

LANDY, M., MICHAEL, G., TRAPANI, R., ACHINSTEIN, B., WOODS, M. W. AND SHEAR, M. J.—(1960) *Cancer Res.*, **20**, 1279.

LEVER, W. F., GURD, F. R. N., UROMA, E., BROWN, R. K., BARNES, B. A. SCHMID, K. AND SCHULTZ, E.—(1951) *J. clin. Invest.*, **30**, 99.

PETERMANN, M. L.—(1961) *Med. Clin. N. Amer.*, **45**, 537.

PIKOVSKI, M., TAL, C., SCHLESINGER, M., AND MARGOLIASH, E.—(1957) *Nature, Lond.*, **180**, 185.

ROTTINO, A., ANGERS, J. AND DOOL, A.—(1962) *Proc. Soc. exp. Biol., N.Y.*, **111**, 699.

SACHTLEBEN, P. AND RUHENSTROTH-BAUER, G.—(1961) *Nature, Lond.*, **192**, 982.

SATO, S., AMIZUKA, T. AND SATO, K.—(1962) *Ibid.*, **193**, 779.

SAXEN, E. AND PENTTINEN, I.—(1961) *J. nat. Cancer Inst.*, **26**, 1367.—(1962) *Acta path. microbiol. scand.*, **54**, 75.

SCHEIDEGGER, J. J.—(1955) *Int. Arch. Allergy*, **7**, 105.

TAKADA, A., SAITO, M., TANINO, J. AND EBATA, K.—(1962) *Saishin Igaku*, **17**, 1218. (An English summary of this paper is given in *Jap. J. of Med.* (1962), **1**, 210.)

WINZLER, R. J.—(1953) *Advanc. Cancer Res.*, **1**, 543.


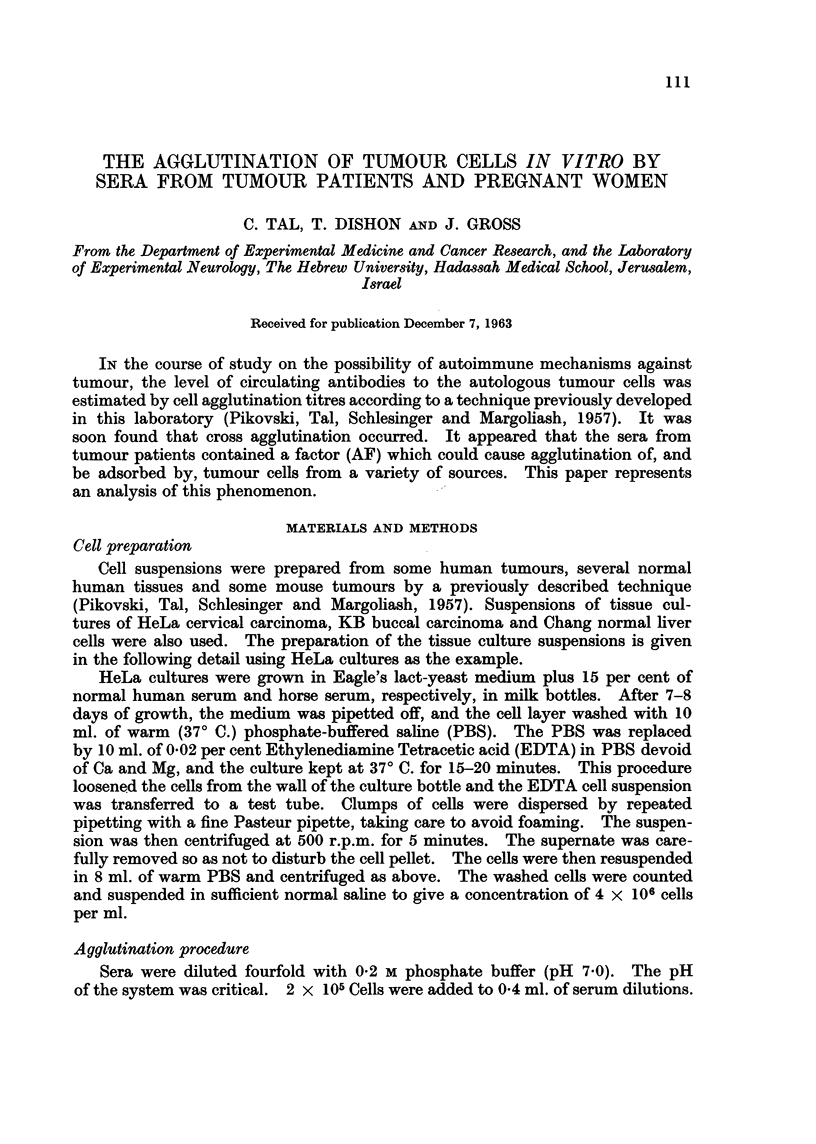

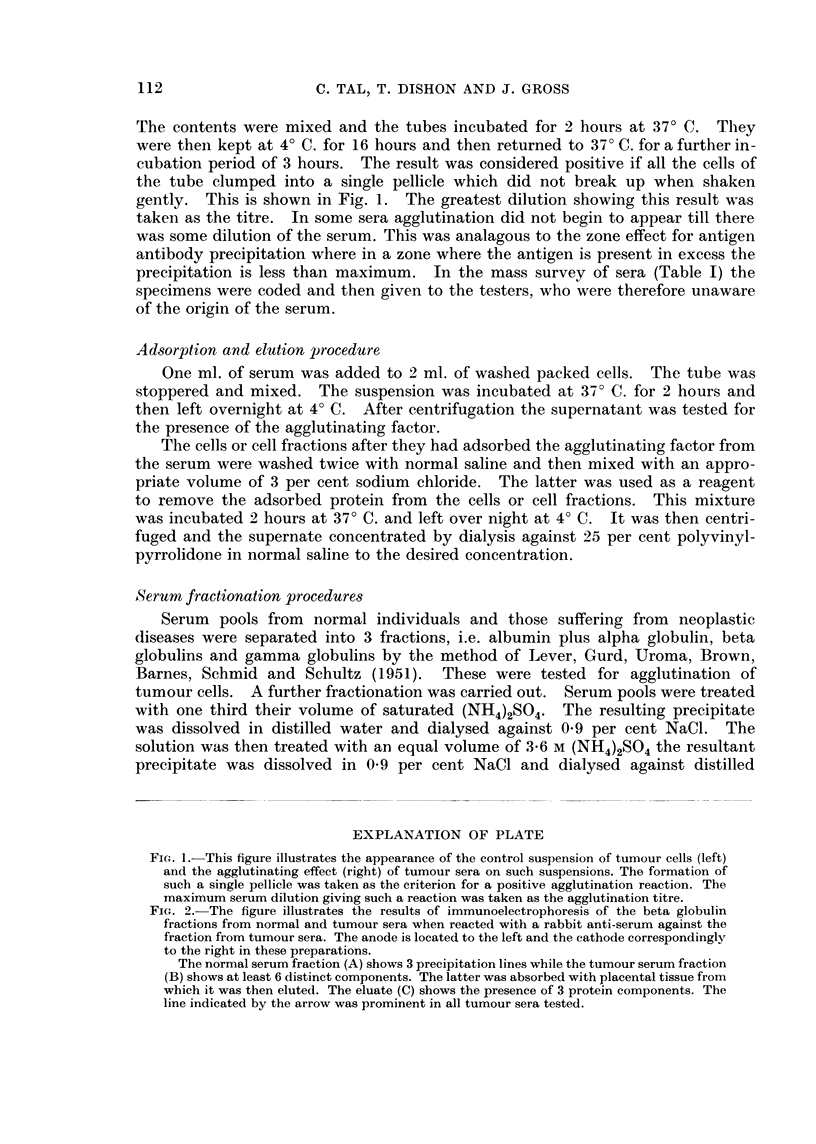

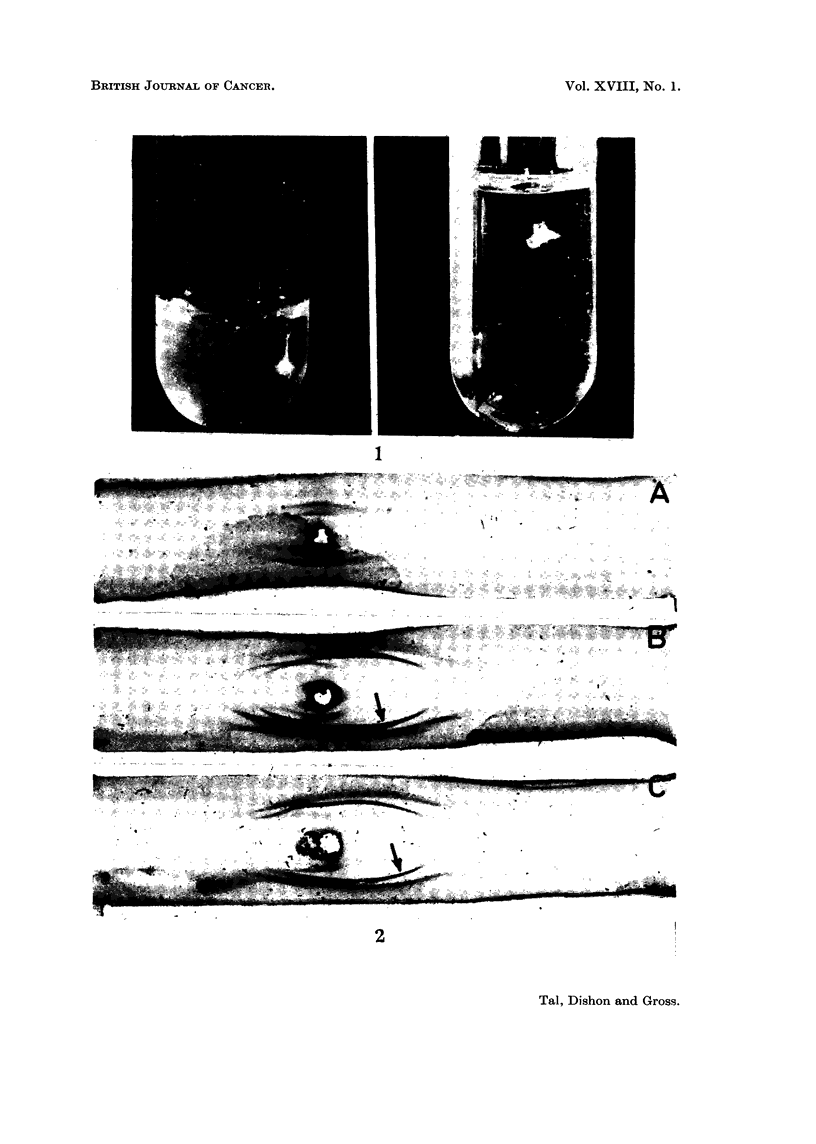

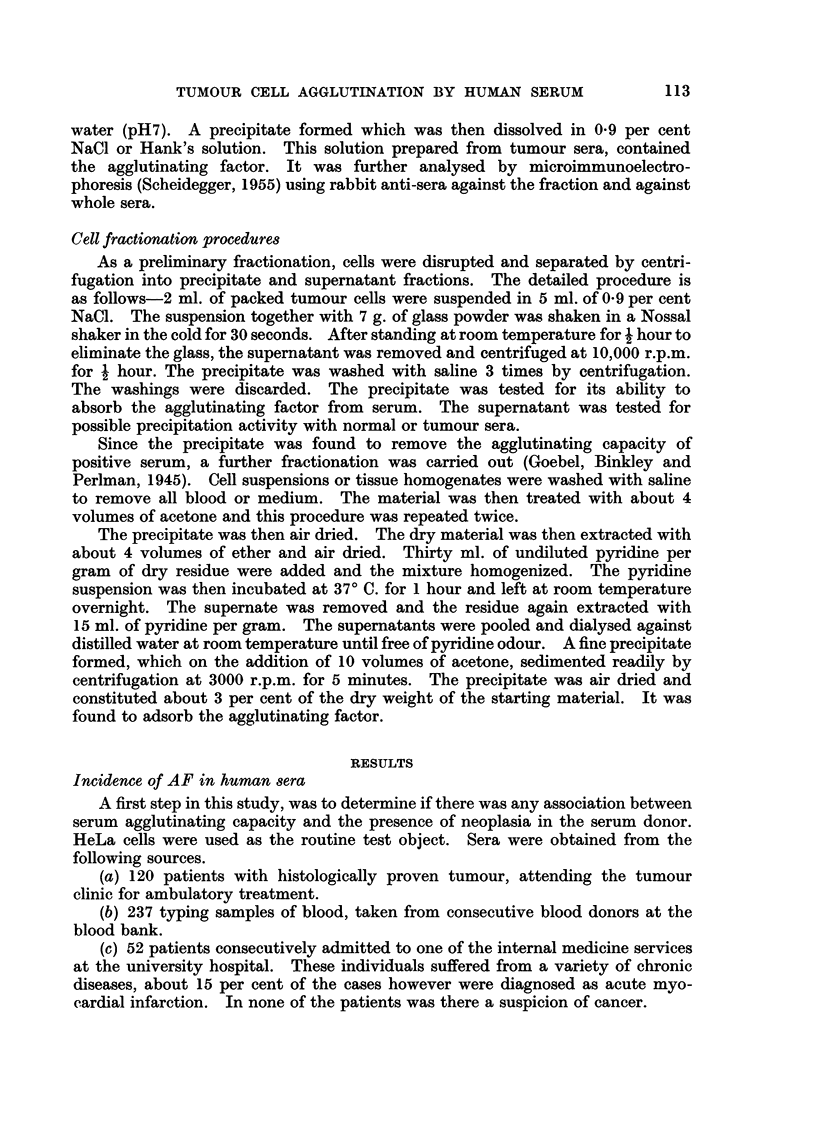

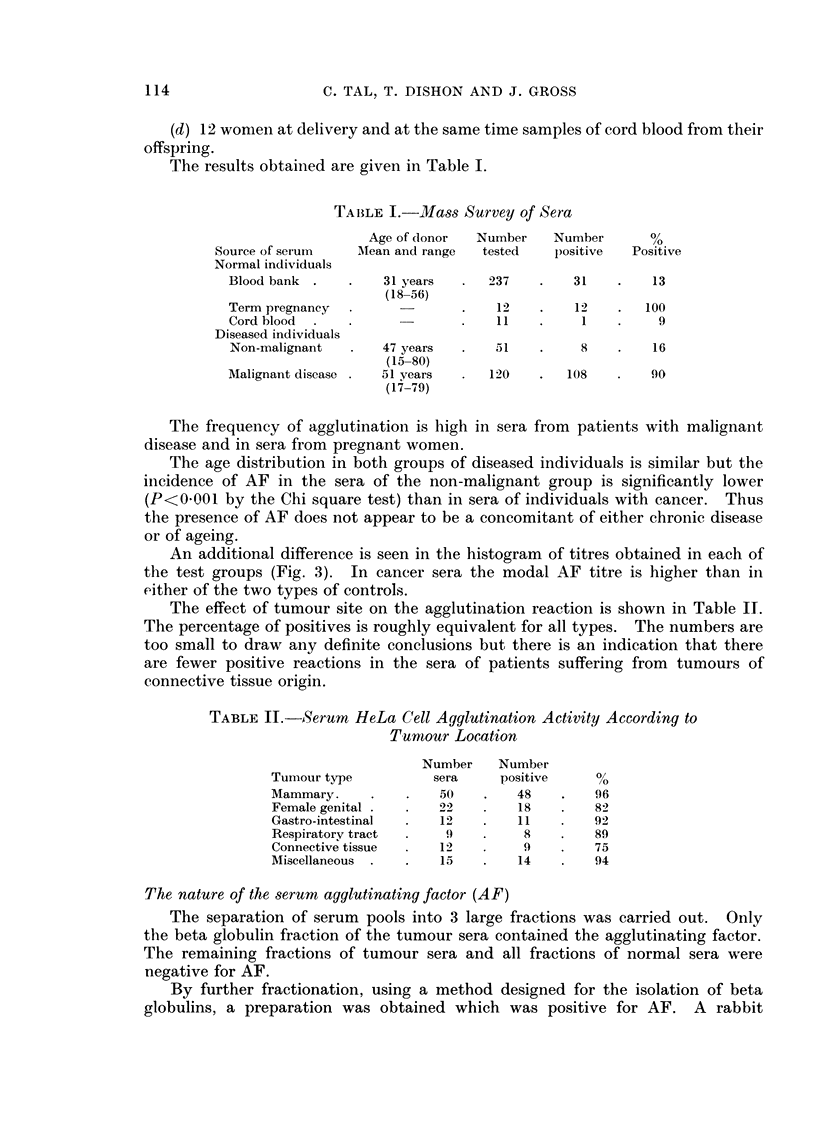

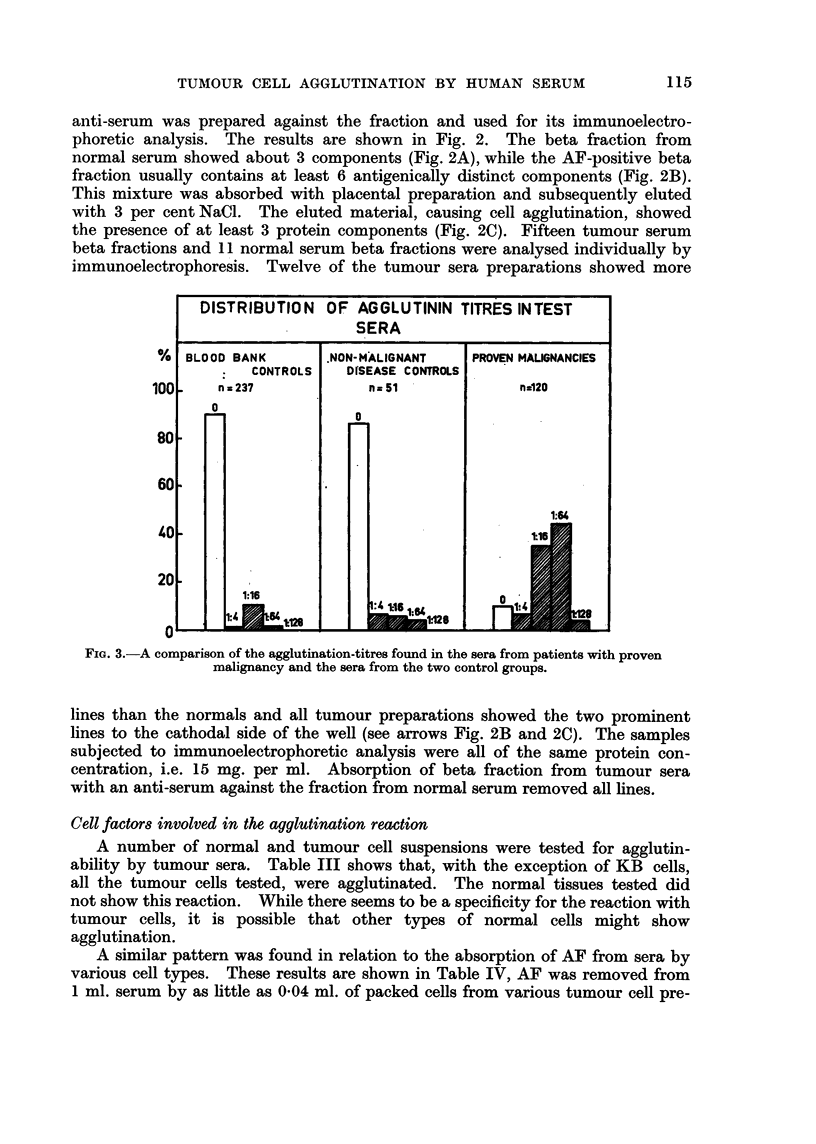

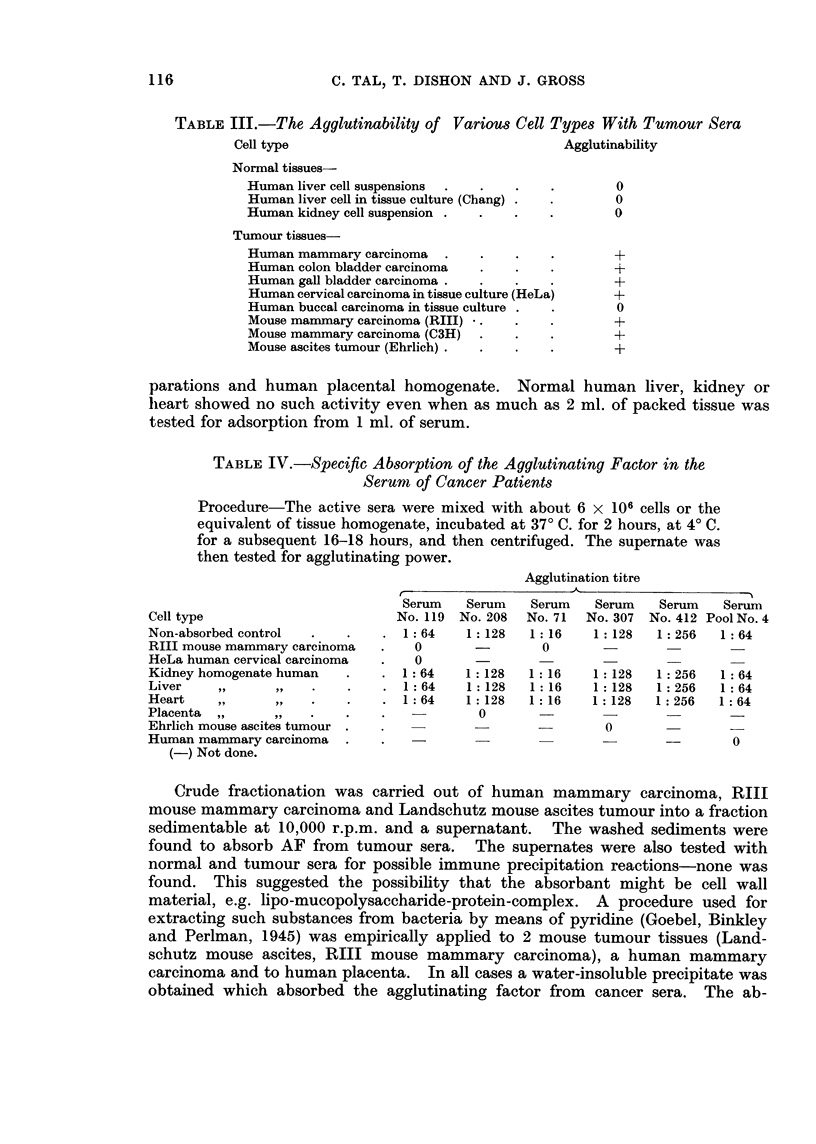

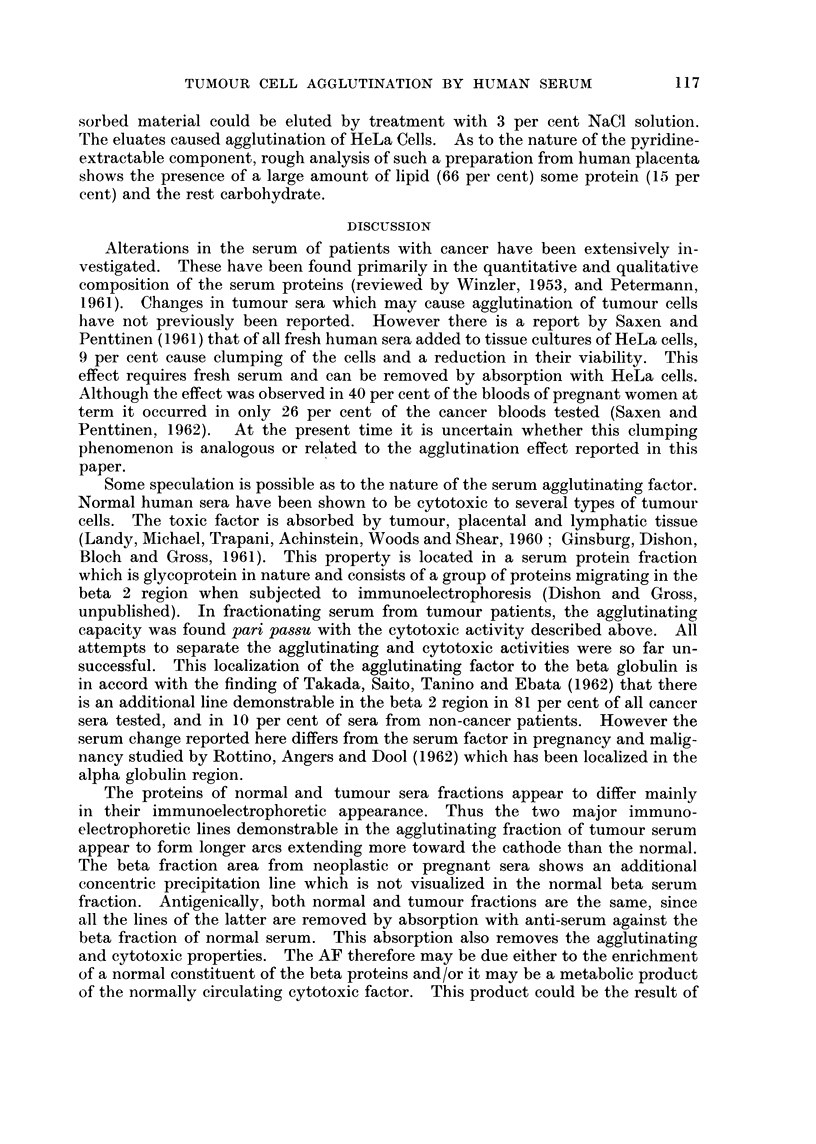

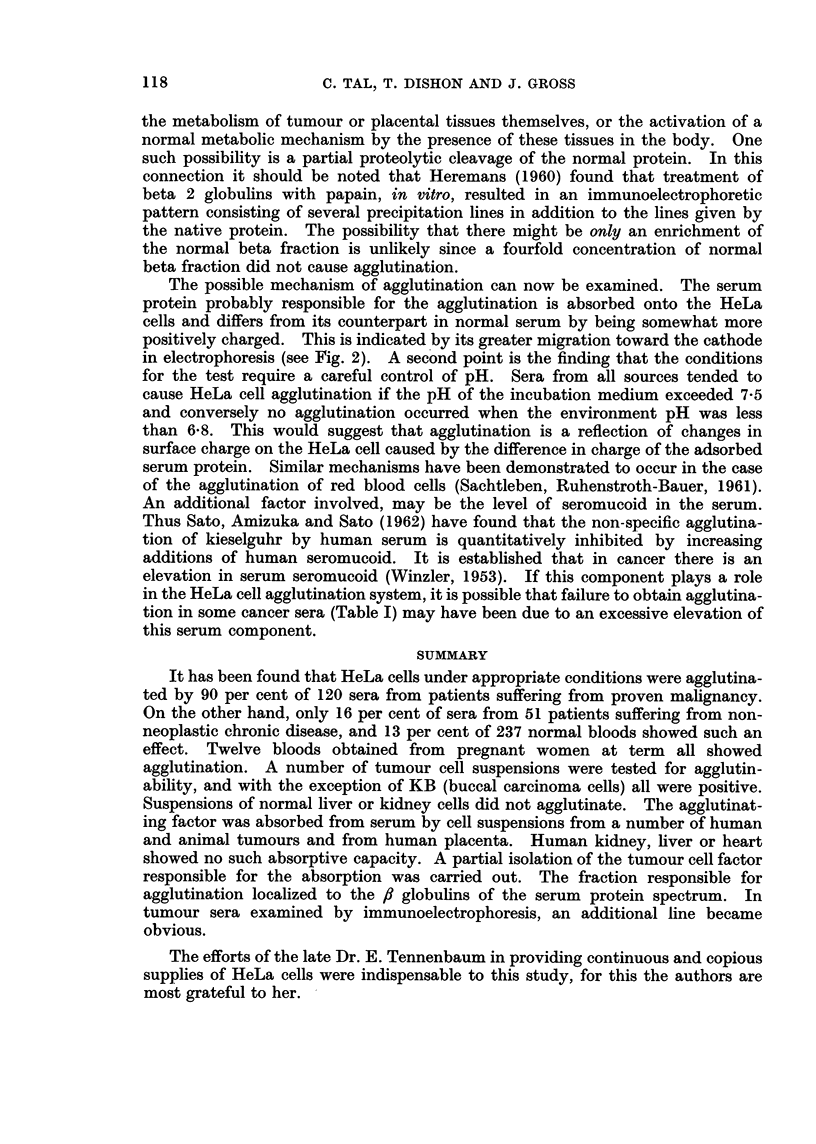

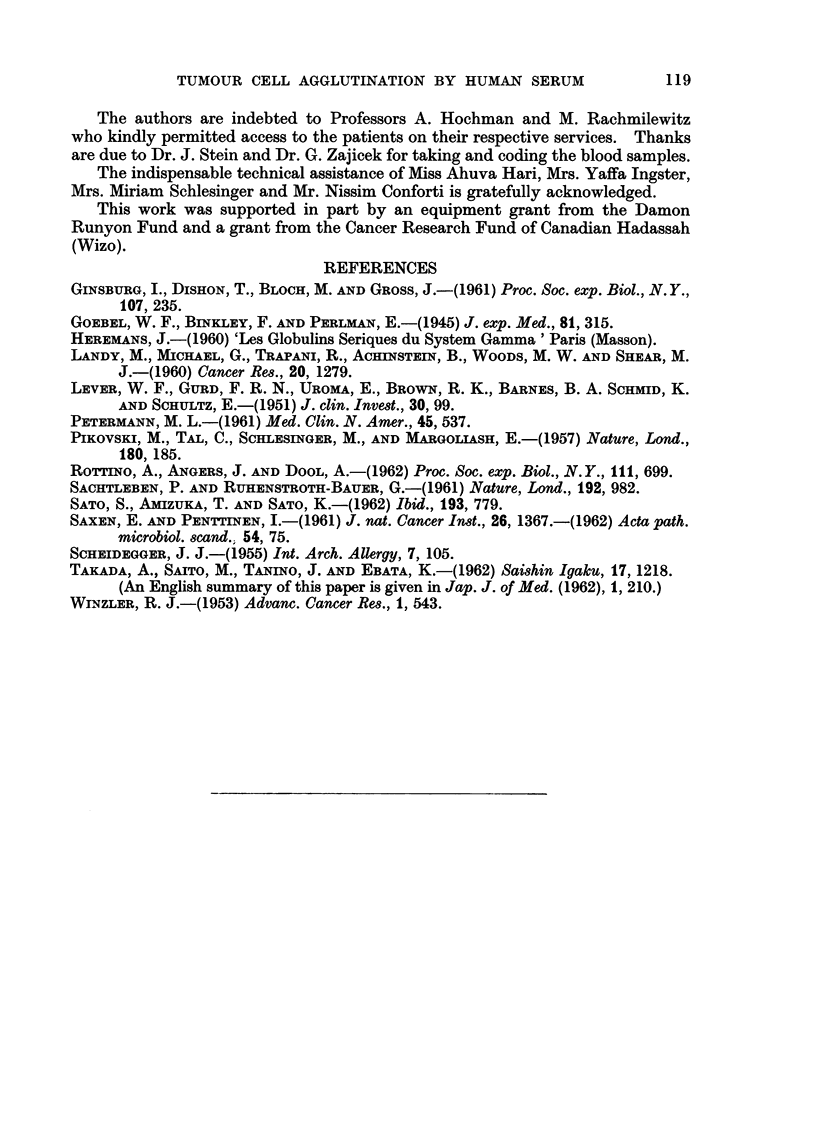


## References

[OCR_00541] GINSBURG I., DISHON T., BLOCK M., GROSS J. (1961). A thermostable cytotoxic factor in normal human serum active against Landschutz ascities tumor cells.. Proc Soc Exp Biol Med.

[OCR_00551] LANDY M., MICHAEL J. G., TRAPANI R. J., ACHINSTEIN B., WOODS M. W., SHEAR M. J. (1960). An antibody-complement system in normal serum lethal to mouse tumor cells.. Cancer Res.

[OCR_00557] PETERMANN M. L. (1961). Plasma protein abnormalities in cancer.. Med Clin North Am.

[OCR_00559] PIKOVSKI M., TAL C., SCHLESINGER M., MARGOLIASH E. (1957). Serological changes concomitant with the growth of mouse tumours in pretreated rats.. Nature.

[OCR_00563] ROTTINO A., ANGERS J., DOOL A. (1962). Demonstration that red blood cell slowing factor found in cancer serum by microelectrophoresis is an alpha1 component.. Proc Soc Exp Biol Med.

[OCR_00564] SACHTLEBEN P., RUHENSTROTH-BAUER G. (1961). Agglutination and the electrical surface potential of red blood cells.. Nature.

[OCR_00565] SATO S., AMIZUKA T., SATO K. (1962). Inhibition of kieselguhr agglutination of serum by seromucoid and pig serum glycoprotein content.. Nature.

